# The impact of immobilized metal affinity chromatography (IMAC) resins on DNA aptamer selection

**DOI:** 10.1007/s00216-014-7937-y

**Published:** 2014-06-13

**Authors:** E. Kowalska, F. Bartnicki, K. Pels, W. Strzalka

**Affiliations:** Department of Plant Biotechnology, Faculty of Biochemistry, Biophysics and Biotechnology, Jagiellonian University, Gronostajowa 7, 30387 Krakow, Poland

**Keywords:** SELEX, IMAC, Aptamers

## Abstract

DNA aptamers are single-stranded oligonucleotides which can form various secondary and tertiary structures. They can recognize a broad range of targets ranging from small molecules, such as ions, vitamins, antibiotics, to high molecular weight structures, including enzymes and antibodies. DNA aptamers are extensively studied as a potential source of new pharmaceutical drugs due to their inexpensive synthesis, low immunogenicity, and high specificity. The commonly used aptamer selection procedure is systematic evolution of ligands by exponential enrichment (SELEX) where the target molecule is immobilized on an appropriate chromatography resin. For peptide/protein targets, immobilized metal affinity chromatography (IMAC) resins are frequently used. There is a broad range of commercially available resins which can be used for IMAC. They are characterized by different metal ions, linker types, and bead materials. In this study, we tested the impact of different IMAC resins on the DNA aptamer selection process during eight SELEX cycles. A histidine-tagged 29 amino acid peptide corresponding to the interdomain connecting loop of human proliferating cell nuclear antigen was used as a selection target. Different resin materials containing the same metal ion (Co^2+^) were tested. Simultaneously, agarose resins containing identical linkers, but different metal ions (Co^2+^, Cu^2+^, Ni^2+^, and Zn^2+^) were analyzed. The results of this study clearly demonstrated the impact of the metal ion and resin material on the DNA aptamer selection progress. The presented data indicate that for successful IMAC resin-based SELEX, the determination of the optimal resin might be crucial.

## Introduction

DNA or RNA aptamers are short single-stranded molecules, from 40 to 100 nucleotides in length, which are able to form unique secondary and tertiary structures. They can recognize many different targets, starting from small molecules such as metal ions and amino acids to large protein complexes or even whole viruses and cells, and bind them with high affinity and specificity [1]. The comparative analysis of antibodies and aptamers shows that they may bind target molecules with similar affinity. Moreover, the latter are characterized by lower immunogenicity and molecular weight. The biological, chemical, and physical properties and relatively low synthesis cost of DNA aptamers make them an interesting source of new tools used in scientific research and clinical diagnostics. Moreover, thanks to their advantages, aptamers are also of great interest as potential modern therapeutic drugs. There is already an example of commercially produced aptamer successfully used in prevention of macular degeneration (anti-VEGF165 aptamer, Macugen) [2].

Aptamers are usually selected using the systematic evolution of ligands by exponential enrichment (SELEX) method which was described in the early 90s of the last century [3, 4]. The classical SELEX technique where DNA aptamers are developed can be divided into four major stages. In the first step, the target molecule is exposed to a single-stranded DNA aptamer library containing usually around 10^14^ different oligonucleotides. The commonly used libraries contain aptamers composed of a 20–60 nucleotide variable region flanked by two regions which serve as sites for primer annealing during PCR-mediated library amplification. Next, unbound oligonucleotides are washed away. Subsequently, DNA fragments bound to the target are amplified using PCR and finally a single-stranded DNA (ssDNA) pool is prepared for the next selection round. Usually, 4–12 selection rounds are sufficient to enrich the aptamer pool in sequences characterized by high specificity and strong affinity to the target molecule. Finally, the selected aptamers are cloned and sequenced. The key step which usually precedes SELEX is target immobilization. Among the different available carriers including nitrocellulose filters, cyanogen bromide-, NHS ester-, and aldehyde-activated supports, immobilized metal resins are often used. Immobilized metal affinity chromatography (IMAC) resin-based SELEX [5] is often used for the selection of DNA aptamers against peptide/protein targets which are bound to resins through a short histidine tag. In the presented study, we have shown that IMAC resin material and metal ions (Co^2+^, Cu^2+^, Ni^2+^, and Zn^2+^) may have an important impact on DNA aptamer selection.

## Materials and methods

### Chemicals

All chemicals used for buffers and solutions were obtained from Sigma-Aldrich (Germany) and Merck (Germany) if not mentioned otherwise.

### Peptide immobilization

The HC-29 peptide, a fragment of human proliferating cell nuclear antigen containing an N-terminal histidine tag (HHHHHHGGGGKLMDLDVEQLGIPEQEYSC) was purchased from LifeTein (USA). The target was dissolved in DMSO to the concentration of 10 mg/mL. Next, 20 μg of peptide were mixed with 2 μL of either cobalt, copper, nickel, and zinc ions immobilized on the agarose beads (Agarose Bead Technologies, ABT, BioMadrid) or cobalt ions immobilized on polystyrene beads (Dynabeads® Life Technologies). The peptide immobilization was performed in BW buffer (17.5 g/L NaCl; 50 mM phosphate buffer NaH_2_PO_4_/Na_2_HPO_4_; 0.1 % (*v*/*v*) Tween 20; pH 7.5) for 1 h at RT with continuous mixing. After incubation, the beads were washed three times with BW buffer to remove unbound peptide and then three times with AS buffer (137 mM NaCl; 12.7 mM KCl; 10 mM Na_2_HPO_4_; 2 mM KH_2_PO_4_; 5 mM MgCl_2_; 0.1 % (*v*/*v*) Tween 20; pH 7.5) to equilibrate the resin before selection. The immobilization of the target was confirmed using 16 % SDS-PAGE followed by Coomassie Brilliant Blue staining.

### SELEX procedure

The ssDNA library and oligonucleotide primers were synthesized by IBA (Germany). The library was comprised of ssDNA fragments containing a 40 nucleotide random region and two 20-nucleotide specific flanking sequences (CATGCTTCCCCAGGGAGATG(N)_40_GAGGAACATGCGTCGCAAAC). One microgram of ssDNA library was mixed with either 20 μg of polystyrene beads or around 1,000 agarose beads (except for the first selection round where 25 μg of synthetic ssDNA library was used) and incubated for 1 h at RT (negative selection). Next, the unbound aptamers were mixed with either 2 μg of polystyrene beads or 1 agarose bead containing target followed by incubation for 1 h at RT. After incubation, the beads were washed six times in AS buffer to remove unbound aptamers and then were stored in 1 μL of AS buffer at 4 °C until use. The PCR was performed in 50 μL of mixture containing 1× PCR buffer (10 mM Tris–HCl, 50 mM KCl, 1.5 mM MgCl_2_, pH 8.3), 500 μM dNTPs, 1 μM forward (5′-CATGCTTCCCCAGGGAGATG-3′) and 1 μM reverse (5′-phosphate-GTTTGCGACGCATGTTCCTC-3′) primers, 2.5 units of Taq polymerase (Thermo Scientific), and 1 μL of selected aptamers as a template. The reaction conditions were 94 °C for 5 min, followed by 24 cycles of 94 °C for 30 s, 55 °C for 30 s, and 72 °C for 30 s; a mastercycler gradient thermocycler (Eppendorf) was used. The PCR product was precipitated using isopropanol. To prepare ssDNA for the next selection round, the DNA strand containing a 5′-phosphate group was digested using exonuclease Lambda. The digestion was performed according to the protocol supplied by the producer with minor modifications. One microgram of dsDNA was digested in a reaction mixture containing 1× buffer (67 mM glycine-KOH, 2.5 mM MgCl_2_, 0.1 % (*v*/*v*) Triton X-100, pH 9.4) and 3.3 units of Lambda exonuclease (Thermo Scientific) followed by incubation for 2 h at 37 °C. Next, the ssDNA was purified using a standard phenol/chloroform extraction procedure and precipitated using isopropanol. The concentration of ssDNA was assessed in 10 % DNA denaturing PAGE using ethidium bromide staining.

### Real-time PCR

The binding of either a selected DNA aptamer pool or the ssDNA library to the target molecule was analyzed by quantitative real-time PCR (qPCR). The beads with immobilized target were incubated with 35 ng of DNA in AS buffer as described in the SELEX procedure section. After the final washing, the beads suspended in 1 μl of AS buffer were directly added to the qPCR mixture. Simultaneously, a reference sample containing 35 ng of DNA in AS buffer was prepared. The qPCR reaction mixture contained 1× HS-PCR Master Mix SYBR (A&A Biotechnology, Poland), 0.5 μM forward, 0.5 μM reverse primers, and as a template: (i) beads with HC-29 peptide after SELEX, (ii) beads without HC-29 peptide after SELEX, or (iii) 0.35 ng of reference DNA. The reaction conditions were 95 °C for 10 min, followed by 35 cycles of 95 °C for 15 s, 53 °C for 30 s, and 72 °C for 30 s. Appropriate negative controls were included. The obtained results were referred to calibration curves based on the used ssDNA library. The binding of tested aptamer pools to the target molecule was expressed as follows: aptamer quantity bound to beads/aptamer quantity present in the reference sample. Next, to compare the change in binding level between tested aptamer pools, the enrichment parameter was calculated as the following ratio: tested aptamer pool binding/initial ssDNA library binding to the target. To analyze the contribution of the tested beads in DNA binding, the specificity parameter was calculated. It was expressed as the following ratio: aptamer pool bound to target-containing beads/aptamer pool bound to empty beads.

## Results and discussion

**Fig. 1:**
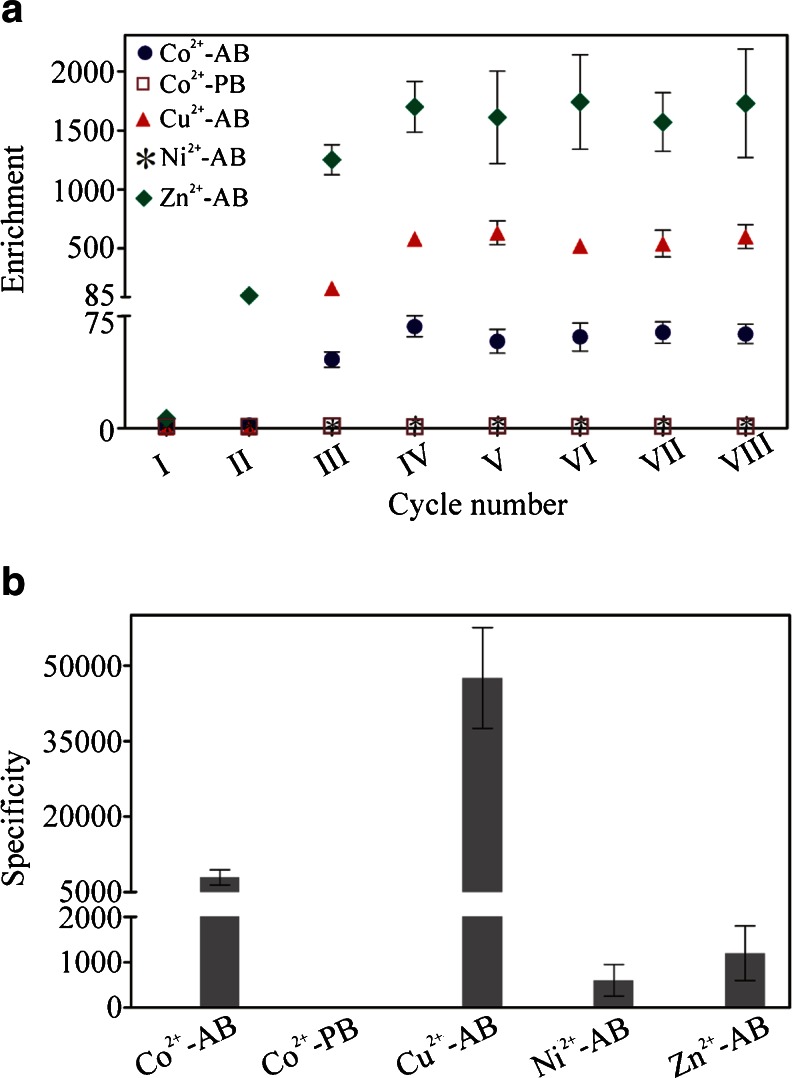
The impact of IMAC resins on SELEX results. The analysis of enrichment and specificity of aptamer pools selected with the help of Co^2+^-, Cu^2+^-, Ni^2+^-, and Zn^2+^-coated agarose beads (−AB) and Co^2+^-coated polystyrene beads (−PB) against the target. **a** The enrichment of aptamer pools after I–VIII selection rounds was expressed as the ratio: binding of tested aptamer pool/binding of initial ssDNA library to molecular target. **b** The specificity of aptamer pools after the eighth SELEX round was expressed as the ratio: binding of aptamer pool to target-containing beads/binding of aptamer pool to empty beads. The presented results are means from three independent experiments. *Error bars* represent the standard deviation

**Fig. 2:**
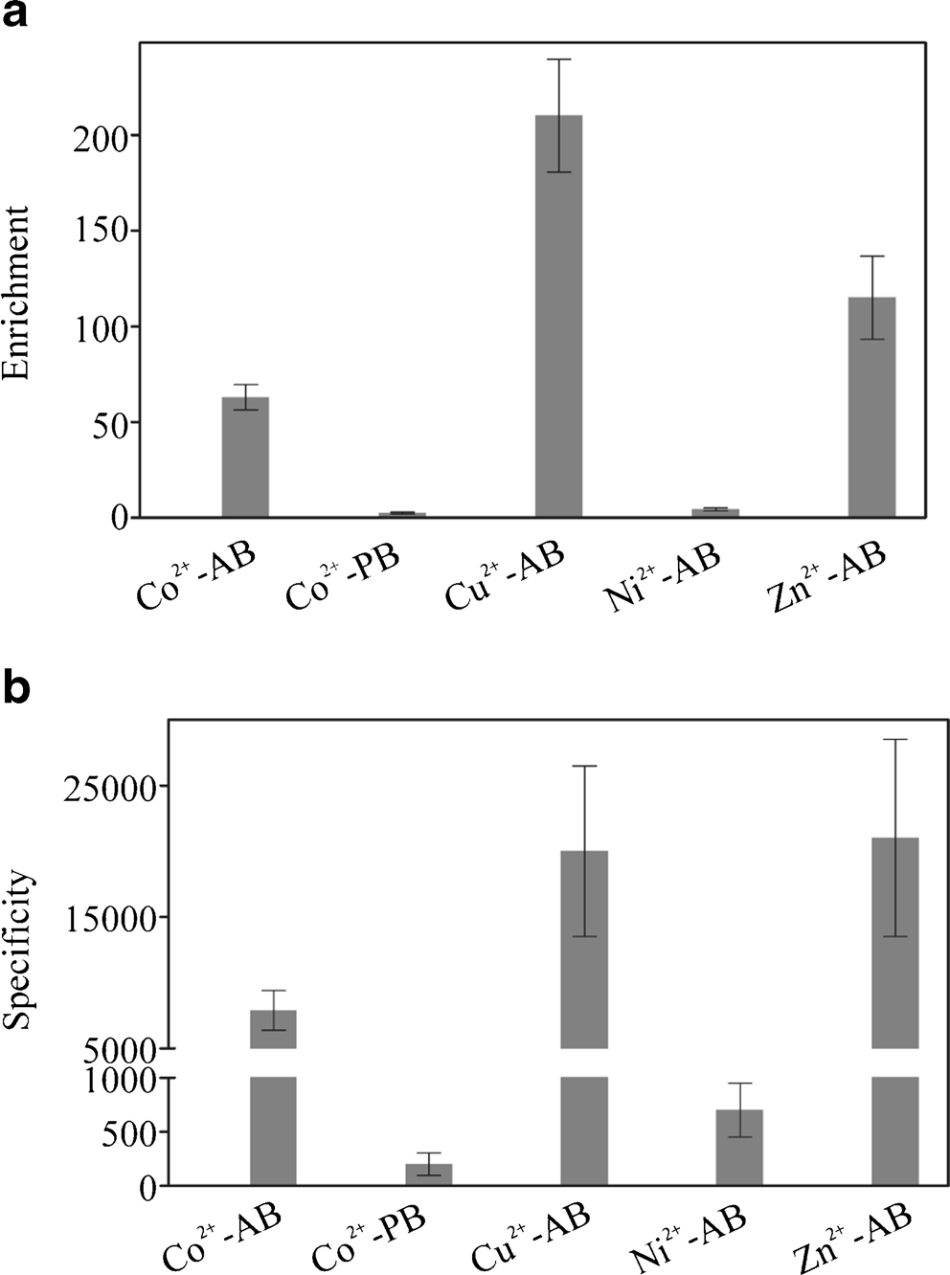
Comparative analysis of agarose and polystyrene resins impact on SELEX results. The enrichment and specificity of aptamer pools after the eighth SELEX round selected with the help of Co^2+^-, Cu^2+^-, Ni^2+^-, and Zn^2+^-coated agarose beads (−AB) and Co^2+^-coated polystyrene beads (−PB) tested using cobalt-coated agarose beads (Co^2+^-AB). **a** The enrichment was expressed as the ratio: binding of tested aptamer pool/binding of initial ssDNA library to molecular target. **b** The specificity was expressed as the ratio: binding of aptamer pool to target-containing beads/binding of aptamer pool to empty beads. The presented results are means from three independent experiments. *Error bars* represent the standard deviation

The first report on SELEX method was published almost 25 years ago [3, 4]. In most cases, the SELEX procedure is performed using target molecules immobilized on an appropriate carrier. The optimal carrier should provide not only a simple and mild target immobilization procedure but also fast and efficient selection of the desired aptamers. Moreover, it should allow undisturbed interaction between the target and aptamer molecules. To obtain high-quality separation of aptamers bound to the target molecule from unbound oligonucleotides, the choice of an adequate carrier is crucial [6]. To compare the carrier effectiveness in the SELEX procedure, a simple method based on real-time PCR can be used where the binding of a selected aptamer pool (e.g., after first, second, third, etc. SELEX round) and the ssDNA library to a target molecule is monitored and compared. Many carriers which are convenient and inexpensive, e.g., nitrocellulose [4] or sepharose [7], were shown to be successfully used in SELEX. Studies of selected target carriers characterized by different features, including nitrocellulose filter, PVDF membrane, octyl-sepharose, and Strataclean^TM^, clearly indicated that they may determine the SELEX result [1]. Among solid-phase carriers, IMAC-based resins are one of the most popular for immobilization of peptide/protein targets containing a short histidine tag. IMAC resins available on the market are characterized by different bead materials, linkers, and metal ions. Taking into account this fact, we studied whether the results of IMAC resin-based SELEX might be dependent on carrier properties. This hypothesis was never tested before. Surprisingly, the obtained results showed significant differences between the tested resins. In the first experiment, cobalt-containing agarose and polystyrene beads to which a short peptide corresponding to the interdomain connecting loop of human proliferating cell nuclear antigen was bound were used as a selection target. Increased binding of selected aptamer pools to the target peptide was detected only when cobalt-coated agarose beads were used (Fig. 1a). The binding level of the analyzed aptamer pool after the second selection round was only about 2.1-fold higher when compared to the ssDNA library. However, after the third and fourth selection round, the enrichment of the tested aptamer pools substantially increased, 46-fold and 68-fold, respectively. The value of enrichment parameter analyzed for aptamer pools after V–VIII SELEX round was not improved in comparison to the aptamer pool after the fourth selection cycle. To evaluate the contribution of empty beads in the binding of tested aptamer pools, the specificity parameter was calculated. This analysis performed for the aptamer pool after the eighth selection round revealed that its binding to target-containing resin was over 7,900-fold higher when compared to empty beads (Fig. 1b). In contrast to these results, when polystyrene beads where used, no significant difference in binding between the ssDNA library and the tested aptamer pools (after I–VIII SELEX rounds) to the target molecule was observed (Fig. 1a). Moreover, the binding of the tested aptamer pool after the eighth selection round to empty beads was around above 1.8-fold weaker than to beads containing our target peptide (Fig. 1b). The results of the performed experiments clearly suggest that the choice of an appropriate IMAC resin might be critical for successful aptamer selection. The observed difference between the tested carriers is not obvious. To clarify which part of the polystyrene beads might be responsible for the observed effect, cobalt-containing polystyrene beads characterized by appropriate bead size and linker should be used. Unfortunately, such resins are not commercially available. Following the above experiments, we postulated that a successful SELEX procedure might be dependent not only on bead material and linker but also the metal ion may play a crucial role. To study this hypothesis, agarose resins characterized by identical linker and different metal ions were used (ABT resins). After the eighth SELEX round, performed on cobalt-, copper-, nickel-, and zinc-coated agarose beads, the selected aptamer pools showed between 4 to over 1,700-fold higher binding to the target than the ssDNA library (Fig. 1a). In the case of copper-containing beads, after the second SELEX round, the selected aptamer pool did not show a significantly higher binding to the target molecule when compared to the ssDNA library. However, for aptamer pools after the third and fourth SELEX round, respectively, more than 160-fold and 580-fold higher binding was observed in comparison to the ssDNA library. The value of enrichment parameter calculated for aptamer pools obtained after V–VIII SELEX round was not significantly improved when compared to aptamer pool after fourth selection cycle. In addition, after the eighth SELEX round, the enrichment of the aptamer pool selected on copper-containing agarose beads was around ninefold higher than of the corresponding aptamer pool obtained after selection on cobalt-coated agarose beads (Fig. 1a). This could be the result of a different quantity of peptide bound to the resin, which might be dependent on metal ion density on the beads. Moreover, the difference in the strength of ligand adsorption between cobalt and copper, which was previously shown when cytochrome c was studied [8], could also have an impact on this result. The contribution of copper-containing agarose beads in binding of the aptamer pool after the eighth SELEX round was insignificant. It was reflected in the almost 48,000-fold higher aptamer binding to beads containing the target peptide than to empty beads (Fig. 1b). In contrast to copper- and cobalt-coated agarose resins, very low enrichment of aptamer pools was observed when nickel-coated agarose resin, commonly used for histidine-tagged protein purification, was applied (Fig. 1a). No significant enrichment of the aptamer pools after the first and second SELEX round was observed. After the eighth SELEX round, merely fourfold higher binding was observed in comparison to the ssDNA library. The binding of this aptamer pool to the target-containing resin was merely over 600-fold higher when compared to empty resin (Fig. 1b). Finally, aptamer pools selected using zinc-containing beads were tested. In comparison to the ssDNA library, the binding level of the aptamer pool after the second SELEX round increased over 100-fold, after the third 1,250-fold, and after the fourth 1,700-fold (Fig. 1a). The analysis of aptamer pools obtained after V–VIII SELEX rounds did not demonstrate significant increase of the enrichment parameter value when compared to aptamer pool after fourth selection round. The binding of the aptamer pool after the eighth SELEX round to target-containing beads was 1,200 higher when compared to empty beads (Fig. 1b). However, at the same time, its binding to empty beads was around tenfold higher in comparison to the corresponding aptamer pools selected using cobalt-, copper-, and nickel-coated agarose beads (data not shown). The binding of the latter to empty beads was comparable with the ssDNA library (data not shown). This suggests that in the aptamer pool selected using the zinc-coated agarose carrier, two fractions of DNA molecules were present: target-specific and resin-specific (possibly zinc specific). Moreover, these data indicate that implemented negative selection procedure was not effective in the case of this resin. This is surprising, taking into account the fact that this technique was shown previously to have a spectacular effect when compared to the classical SELEX procedure [9]. To evaluate which of the tested resins was the most suitable for the selection of aptamers against our target, in the final experiment, we used a cobalt-coated agarose resin to compare the enrichment and specificity of aptamer pools after the eighth SELEX round selected on Co^2+^-, Cu^2+^-, Ni^2+^-, and Zn^2+^-coated agarose beads and Co^2+^-coated polystyrene beads (Fig. 2). This experiment was in agreement with our previous results and showed that optimal enrichment and specificity of aptamers selected against our target was achieved when either copper- or cobalt-coated agarose resins were used. Moreover, in case of the aptamer pool selected on zinc-coated agarose beads, the specificity of binding to the target on cobalt-coated agarose resin was around 17 times higher (Fig. 2b) than on zinc-coated agarose resin (Fig. 1b). This was due to the low binding level of the aptamer pool after the eighth SELEX round, selected with the help of zinc-coated agarose beads, to empty cobalt-coated agarose resin (data not shown). The above result supports our hypothesis that this aptamer pool was composed of two DNA molecule fractions: one specific for our target and the second for the resin.

## Conclusions

Our studies significantly complement the reports that address SELEX methodology and suggest that to reduce the time necessary for successful IMAC resin-based aptamer selection, different types of resins should be simultaneously tested. This information should be especially useful and considered when an almost fully automated SELEX procedure is planned.

## Abbreviations

IMAC: Immobilized metal affinity chromatography
ssDNA: Single-stranded DNA
SELEX: Systematic evolution of ligands by exponential enrichment
